# *Lactobacillus salivarius* HHuMin-U Activates Innate Immune Defense against Norovirus Infection through TBK1-IRF3 and NF-κB Signaling Pathways

**DOI:** 10.34133/research.0007

**Published:** 2022-12-19

**Authors:** Da Hyun Kim, Minju Jeong, Jae Hwan Kim, Joe Eun Son, John J. Y. Lee, Sang-jun Park, Juyeon Lee, Minwoo Kim, Jong-Won Oh, Myeong Soo Park, Sanguine Byun

**Affiliations:** ^1^ Department of Biotechnology, Yonsei University, Seoul 03722, Republic of Korea.; ^2^ Department of Agricultural Biotechnology, Seoul National University, Seoul 08826, Republic of Korea.; ^3^ Program in Developmental and Stem Cell Biology, The Hospital for Sick Children, Toronto, Ontario, Canada.; ^4^ Research Center, BIFIDO Co. Ltd., Hanam 12930, Republic of Korea.

## Abstract

The composition of commensal bacteria plays a critical role in controlling immune responses in the intestine. Studies have shown that specific bacterial strains may have the capacity to enhance host immune defense against gastrointestinal viral infections. While norovirus is known to be the most common cause of gastroenteritis, leading to an estimated 200,000 deaths every year, identification of bacterial strains with protective effects against norovirus infection remains elusive. Here, we discovered *Lactobacillus salivarius* HHuMin-U (HHuMin-U) as a potent antiviral strain against norovirus infection. HHuMin-U significantly suppressed murine norovirus replication and lowered viral RNA titers in macrophages. The transcriptome sequencing (RNA sequencing) analysis revealed that HHuMin-U markedly enhanced the expression level of antiviral interferon-stimulated genes compared to mock treatment. HHuMin-U treatment dose-dependently induced type I interferons (IFN-α and IFN-β) and tumor necrosis factor-α production in mouse and human macrophages, promoting antiviral innate responses against norovirus infection. Investigation on the molecular mechanism demonstrated that HHuMin-U can activate nuclear factor κB and TANK-binding kinase 1 (TBK1)–interferon regulatory factor 3 signaling pathways, leading to the phosphorylation of signal transducer and activator of transcription 1 and signal transducer and activator of transcription 2, the key mediators of interferon-stimulated genes. Finally, oral administration of HHuMin-U increased IFN-β levels in the ileum of mice and altered the gut microbiome profile. These results suggest the species/strain-specific importance of gut microbial composition for antiviral immune responses and the potential use of HHuMin-U as a probiotic agent.

## Introduction

Norovirus is the most common cause of epidemic gastroenteritis disease, accounting for 18% of diarrheal diseases worldwide and causing an estimated 200,000 deaths every year [1,2]. The primary mode of transmission is the fecal–oral spread. In addition to person-to-person contact or airborne spread, the consumption of contaminated food has been identified as the main cause of transmission [3]. Contact with human fecal matter at the source, or unsanitary manipulation by a food handler shedding the virus, can contaminate food items such as raw fruits, leafy greens, and oysters [4]. Noroviruses are especially contagious because of their low infectious dose (approximately 10 to 100 viral particles) and their ability to tolerate a broad range of temperatures [5]. Consequently, these viruses have been implicated in numerous cases of large-scale food-borne outbreaks, particularly in group meal service facilities such as cruise ships, hospitals, and nursing homes, causing tremendous social and economic costs [6–9]. Although symptoms are usually self-limited for healthy people, severe outcomes might occur among immunocompromised patients, young children, and elderly populations [10,11]. Despite recent attempts to the development of norovirus vaccines, there have not been any approved vaccines or drugs for clinical use. As a result, there is an unmet need for therapeutic measures against norovirus infection.

The innate immune system is the first line of defense against viral infections. Within the multiple layers of immunological responses, type I interferons (IFNs) play a critical role in antiviral host defense by inhibiting viral replication and potentiating adaptive immune responses via both direct and indirect mechanisms [12]. Innate immunity is highly essential in limiting norovirus infection. The first murine norovirus (MNV) was identified in immunodeficient mice lacking signal transducer and activator of transcription 1 (STAT1), the downstream transcription factor in type I signaling pathways, highlighting the importance of innate immunity in MNV control [13]. Ifnar1^−/−^ mice succumb to MNV infection despite enhanced adaptive immunity, and IFN regulatory factor 3 (IRF3) and IRF7-mediated type I IFN production has been shown to restrict MNV replication [14,15]. In addition, norovirus infection is recognized to cause prolonged and lethal illness in immunocompromised patients [16–18]. Recent studies have also revealed the importance of type I and type III IFN responses for restricting human norovirus replication [19–21]. These results strongly demonstrate that innate immunity including type 1 IFN responses is indispensable in limiting norovirus infection.

Numerous strains of bacteria reside in the gastrointestinal tract, and the crosstalk between commensal bacteria and intestinal immune cells plays an essential role in controlling immune responses [22]. A study showed that mice raised in a complete germ-free environment display underdeveloped lymphoid tissues in the gut compared with specific pathogen-free conditions, showing the effect of intestinal microflora on immune systems [23]. Intestinal commensal bacteria activate Peyer’s patches via Toll-like receptor (TLR) pathways to induce the secretion of antimicrobial peptides, and they are critical for the clearance of enteric pathogen infections [24,25]. Certain strains of lactic acid bacteria have been reported to exert protective effects against enteric viruses including rotavirus and transmissible gastroenteritis virus [26,27]. In addition, the intestinal microbiome influences norovirus infections. A cohort study demonstrated that changes in microbiota profiles were observed in infants following norovirus infection [28]. The role of commensal bacteria in norovirus infection is further supported by the evidence that compositional differences in the gut microbiome determine the host’s susceptibility to symptomatic norovirus infection [29]. However, it is still unknown which bacterial strain contributes to the resistance of the host against norovirus infection.

Bacteria have been traditionally used to increase the shelf life of food materials through fermentation [30]. However, recently, the development of functional foods containing microbes with health-promoting effects referred to as probiotics has become a major topic of interest. Concurrently, accumulating evidence suggests that specific strains of probiotics are effective in ameliorating diverse disorders such as colitis, hyperlipidemia, and allergies [31–33]. Because their efficacies differ depending on the strains, identifying bacterial strains on the basis of their bioactivity can be beneficial. In this regard, we screened a collection of lactic acid bacteria for their antiviral capacity and discovered *Lactobacillus salivarius* HHuMin-U (HHuMin-U) as a potent antiviral strain against norovirus infection. We further show that inhibition of norovirus infection is related to the production of type I IFNs (IFN-α and IFN-β) and tumor necrosis factor-α (TNF-α) induced by HHuMin-U, leading to the induction of antiviral ISGs. Furthermore, orally administered HHuMin-U exerted upregulation of IFN-β levels in the ileum of mice. Overall, this study highlights the role of HHuMin-U in boosting the intestinal innate immune system against norovirus infection.

## Results

### HHuMin-U inhibits norovirus replication and prevents viral induced apoptosis

**Fig. 1.**
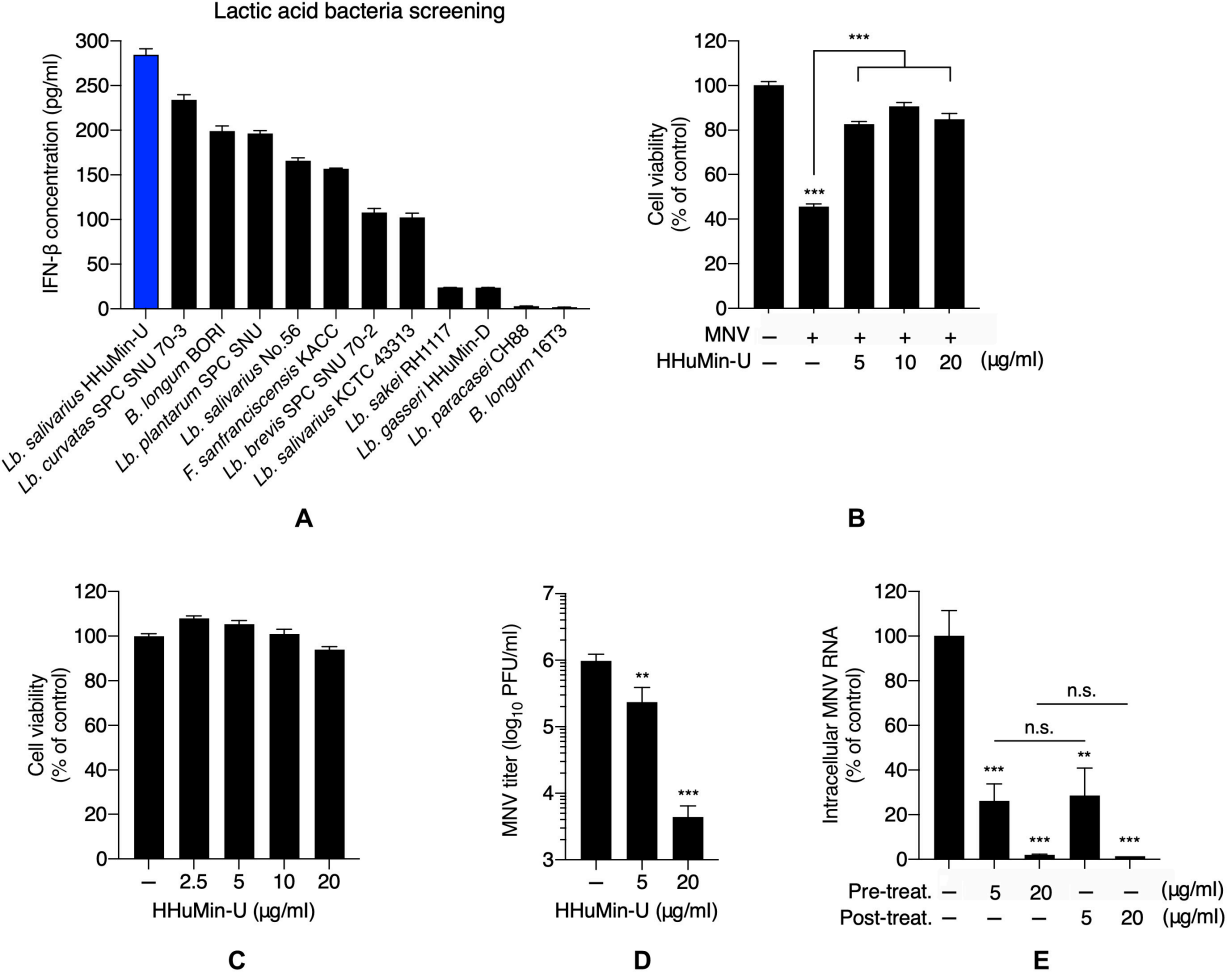
Antiviral activity of *Lb. salivarius* HHuMin-U against murine norovirus. (A) Twelve lactic acid bacteria strains (heat-inactivated) were added to RAW264.7 cells for 24 h. Interferon-β (IFN-β) concentrations in culture media were measured using ELISA. (B) RAW264.7 cells were infected with MNV (MOI of 0.05) and treated with indicated concentrations of HHuMin-U. Cell viability was quantified using the SRB assay at 24 h postinfection. (C) Cell viability was measured using the SRB assay after RAW264.7 cells were treated with HHuMin-U for 48 h. (D and E) MNV (MOI of 0.05)-infected RAW264.7 cells were treated with the indicated concentration of HHuMin-U. (D) MNV titers after 24 h postinfection were determined using a plaque-forming assay. PFU, plaque-forming unit. (E) Intracellular viral RNA titers were quantified by RT-qPCR. Data are shown as mean ± SD. **P < 0.01 and ***P < 0.001; n.s., not significant.

To identify candidate bacterial strains with antiviral potency, a screening was conducted using various lactic acid bacteria. Considering the important role of IFN-β in antiviral innate immunity, we measured the capability to produce IFN-β in various bacterial strains, and HHuMin-U was selected as the primary antiviral candidate (Fig. 1A).

We used MNV as a surrogate for human norovirus to test the antiviral activity of HHuMin-U. MNV infection in RAW264.7 is known to result in extensive cytopathic effect followed by apoptosis [34]. MNV infection at 0.05 MOI (multiplicity of infection) reduced cell viability to 45.5% compared to mock-treated control. HHuMin-U treatment recovered the decreased survival rate up to 90.3%, suggesting that HHuMin-U boosted host cell resistance against MNV (Fig. 1B). Notably, HHuMin-U did not affect cell viability in RAW264.7 at the concentrations tested (Fig. 1C). Furthermore, on the basis of the plaque-forming assay, HHuMin-U treatment at 20 μg/ml led to a 220-fold reduction in viral load (Fig. 1D).

After observing the antiviral activity of HHuMin-U, we decided to determine which step HHuMin-U is blocking during MNV infection. The RNA copy number of MNV was measured by real-time quantitative polymerase chain reaction (RT-qPCR) to calculate the intracellular MNV RNA levels. Posttreatment of HHuMin-U after MNV infection lowered the viral load by 98.7%. Pretreatment of HHuMin-U before MNV infection did not further decrease this level (Fig. 1E), showing comparable inhibitory effect to the posttreatment group. These results suggest that HHuMin-U is primarily involved in enhancing the defense mechanism of host cells against MNV infection, rather than blocking the entry steps of viral infection.

### HHuMin-U stimulates the transcription of a wide range of IFN-stimulated genes (ISGs)

**Fig. 2.**
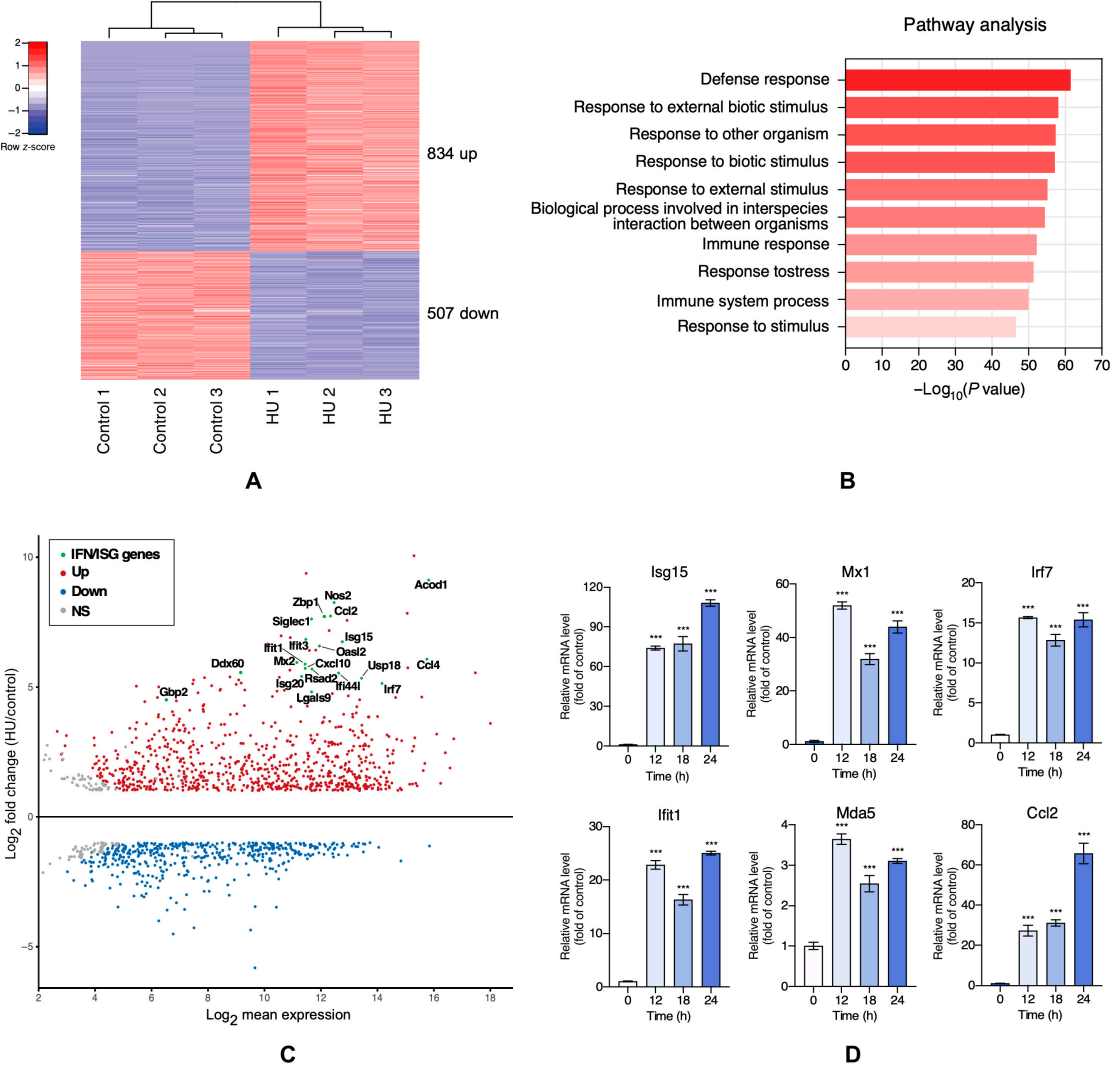
Effect of HHuMin-U (HU) treatment on the antiviral immune responses in macrophages. (A) Heatmap of significant differentially expressed genes (DEGs) in each sample treatment following 12 h of HHuMin-U treatment (log*_2_*(fold change) > 1, adjusted *P* < 0.05). (B) Clustered results of the GO analyses of DEGs in HHuMin-U-treated RAW264.7. The bar graph represents the enrichment scores (−log_10_(*P* value)) of the top 10 significantly enriched GO terms in biological processes. (C) Scatterplot of the results of DEGs analysis with mean expression plotted on the *x* axis and corresponding fold change plotted on the *y* axis for each gene. (D) Validation of RNA sequencing analysis by quantifying transcriptomic changes of ISGs in RAW264.7 after HHuMin-U treatment using RT-qPCR. Data are shown as mean ± SD. ***P < 0.001

We conducted transcriptome profiling using RNA sequencing to elucidate the antiviral mechanism of HHuMin-U. RNA sequencing was performed on 3 independent replicates of HHuMin-U-treated and mock-treated RAW264.7 cells. The transcriptome of macrophages treated with HHuMin-U had 834 upregulated and 507 downregulated genes, and a hierarchical clustering heatmap of all the significant differentially expressed genes (DEGs) across the replicates confirmed the strong influence of HHuMin-U treatment as the source for differences in gene expression (Fig. 2A). Intriguingly, following gene ontology (GO) classifications and Kyoto Encyclopedia of Genes and Genomes pathways analysis, the top 10 enriched pathways by HHuMin-U treatment were related with immune and defense responses, including “defense response” (−log_10_(*P* value) = 61.48), “response to external biotic stimulus” (−log_10_(*P* value) = 58.04), “response to other organism” (−log_10_(*P* value) = 57.37), and “innate immune response” (−log_10_(*P* value) = 52.25) (Fig. 2B). In contrast, there was no significantly enriched pathway in mock-treated samples (data not shown). These results suggest that HHuMin-U treatment could induce antiviral defense response in macrophages.

IFN-stimulated genes (ISGs), a set of genes induced by IFN production, are considered the ultimate antiviral effectors, establishing the host’s antiviral state [35]. According to the MA plot distribution of DEGs, genes that were identified as ISGs by previous studies including Acod1, Nos2, Ccl2, Zbp1, Siglec1, Ifit3, Isg15, Oasl2, Ccl4, Mx2, Ifit1, Cxcl10, Rsad2, Ddx60, Ifi44l, Usp18, Irf7, Lgals9, and Gbp2 exhibited increased gene expression with HHuMin-U treatment (Fig. 2C) [35–37]. To validate transcriptomic changes identified from the RNA sequencing data, 6 genes that encode key antiviral ISGs were selected, and the expression changes were examined by RT-qPCR. ISGs including Isg15, Mx1, Irf7, Ifit1, Mda5, and Ccl2 showed an increase in the level of mRNA expression over the time examined (Fig. 2D). Overall, we confirmed that HHuMin-U treatment promotes the antiviral status of the host cell by stimulating the transcription of a diverse set of ISGs.

### HHuMin-U induces the production of type I IFNs, IFN-α and IFN-β, and TNF-α

As transcriptomic analysis demonstrated that HHuMin-U treatment upregulated the ISG levels, we speculated that type I IFNs, which trigger the expression of ISGs, may be responsible for the antiviral activity of HHuMin-U. Although TNF-α does not display direct antiviral activity alone, it is known to promote IFN-β production and mediate an autocrine loop that induces the expression of ISGs [38]. Therefore, we examined whether HHuMin-U can induce the production of type I IFNs, IFN-α and IFN-β, and TNF-α in macrophages.

**Fig. 3.**
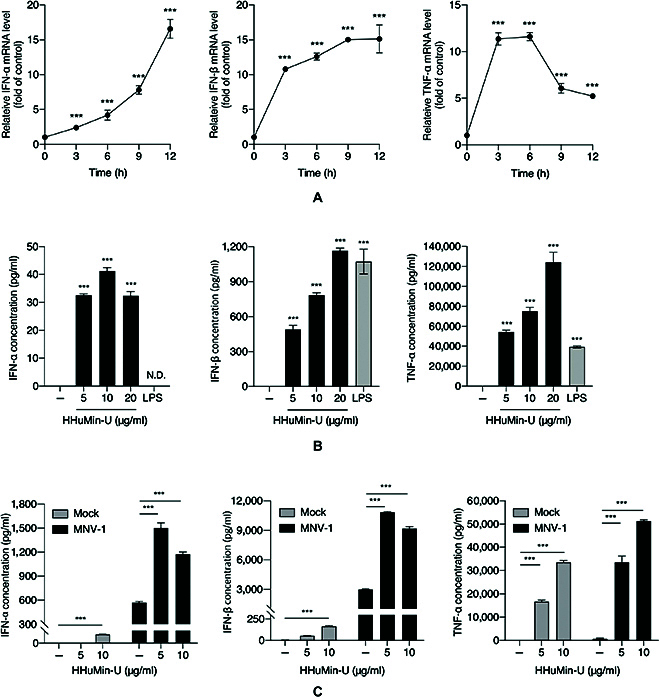
Induction of type I IFNs, IFN-α and IFN-β, and TNF-α production by HHuMin-U in macrophages. (A) RAW264.7 cells were treated with HHuMin-U (20 μg/ml). The mRNA levels of IFN-α, IFN-β, and TNF-α at the indicated time points were determined by RT-qPCR. (B) The cells were treated with increasing concentrations of HHuMin-U or lipopolysaccharide (LPS) (200 ng/ml) for 24 h. The protein levels of IFN-α, IFN-β, and TNF-α in RAW264.7 were analyzed by ELISA. (C) RAW264.7 cells, which were either mock infected or MNV infected (MOI of 0.05) were treated with the indicated concentrations of HHuMin-U, and the protein levels of IFN-α, IFN-β, and TNF-α were measured by ELISA. Data are shown as mean ± SD. ***P < 0.001; ND, not detected.

Although expression kinetics varies, HHuMin-U increased IFN-α, IFN-β, and TNF-α mRNA levels in RAW264.7 cells at the indicated time points (Fig. 3A). HHuMin-U treatment also induced IFN-α, IFN-β, and TNF-α protein levels in RAW264.7 cells (Fig. 3**(b)**). We also tested the effect of HHuMin-U on human-originated macrophage-like cells. Consistently, IFN-α, IFN-β, and TNF-α mRNA expressions were increased in human macrophage-like THP-1 cells by HHuMin-U treatment (Fig. S1).

We further investigated the effect of HHuMin-U on IFN-α, IFN-β, and TNF-α production under MNV infection conditions. While MNV infection induced IFN-α, IFN-β, and TNF-α to some extent, co-treatment with HHuMin-U further elevated these levels (Fig. 3C). This result demonstrates that HHuMin-U primes macrophages to boost type I IFNs and TNF-α production in MNV-infected cells, enhancing host defense mechanisms against viral infections.

### HHuMin-U activates NF-κB and TBK1–IRF3 signaling, leading to STAT1/2-mediated ISG expression

**Fig. 4.**
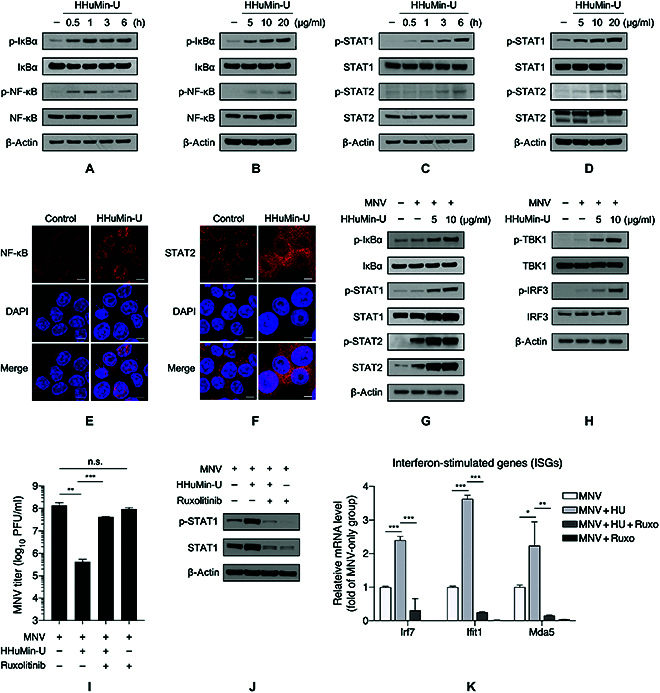
Activation of NF-κB, TBK–IRF3, and STAT1/2 signaling pathways by HHuMin-U. (A and C) RAW264.7 cells were treated with HHuMin-U (20 μg/ml) for 0.5, 1, 3, and 6 h. Protein expression levels of phosphorylated and total (A) IκB and NF-κB as well as (C) STAT1 and STAT2 in cell lysates were determined by immunoblotting. (B and D) RAW264.7 cells were treated with the indicated concentrations of HHuMin-U. Protein expression levels of phosphorylated and total (B) IκB and NF-κB as well as (D) STAT1 and STAT2 were determined by immunoblotting. (E and F) The effect of HHuMin-U on (E) NF-κB and (F) STAT2 translocation was examined by immunofluorescence. Scale bars, 5 μm. (G and H) Mock-infected or MNV-infected (MOI of 0.05) RAW264.7 cells were treated with HHuMin-U. Immunoblotting analysis for protein levels of phosphorylated and total (G) IκB, STAT1, and STAT2 as well as (H) TBK1 and IRF3 in cell lysates. β-Actin was used as a loading control. (I to K) RAW264.7 cells were treated or untreated with ruxolitinib (10 μM) and then infected with MNV (MOI of 0.05). HHuMin-U (20 μg/ml) was treated for 24 h and (I) MNV titers were measured by plaque-forming assay, (J) protein levels of phosphorylated and total STAT1 were measured by immunoblotting, and (K) mRNA levels of ISGs were quantified by RT-qPCR. Data are shown as mean ± SD. *P < 0.05, **P < 0.01, and ***P < 0.001.

We further examined the molecular mechanism of HHuMin-U for its antiviral activity. Having found the capability of HHuMin-U to induce IFN-α, IFN-β, and TNF-α in macrophages, we examined the effect of HHuMin-U on nuclear factor κB (NF-κB) and IRF3 and their upstream regulators as they are reported to be the major transcription factors for antiviral cytokines and type I IFN genes, respectively. In addition, accumulating evidence suggests that the NF-κB signaling pathway activates IRFs and contributes to type I IFN gene expression [39]. As shown in Fig. 4A, HHuMin-U induced inhibitor of NF-κBα (IκBα) and NF-κB activation, peaking at 3 h after HHuMin-U treatment. HHuMin-U also dose-dependently enhanced phosphorylation of IκBα and NF-κB (Fig. 4B). Upon binding of type I IFNs to the IFN-α/β receptor (IFNAR), the Janus kinase (JAK)–STAT signaling pathway is activated to form an ISG factor 3 complex, which consists of STAT1, STAT2, and IRF9, and directly triggers the transcription of antiviral ISGs [40]. Therefore, we analyze whether HHuMin-U induces phosphorylation of STAT1 and STAT2. Treatment of HHuMin-U dose-dependently increased STAT1 and STAT2 phosphorylation, peaking at 6 h after treatment (Fig. 4C and D). In accordance with the findings from the signaling analysis, HHuMin-U induced the translocation of NF-κB and STAT2 from the cytoplasm to the nucleus (Fig. 4E and F). This suggests that HHuMin-U can ultimately exert a protective effect against MNV infection through the expression of ISGs.

To elucidate the role of HHuMin-U in the context of viral infection, we examined the same signaling pathways after MNV infections. The phosphorylation levels of IκB, STAT1, and STAT2, which were induced by MNV, were further augmented by HHuMin-U treatment (Fig. 4G). In addition, HHuMin-U dose-dependently elevated the phosphorylation of TBK1 and IRF3, the direct transcriptional factor for IFN-β, in macrophages (Fig. 4H).

We further sought to confirm whether IFN-α/β-driven induction of ISGs is the major mode of action of HHuMin-U in limiting viral replication. As JAK-STAT pathway is known to act as a key downstream effector of IFN-α/β response in upregulating ISGs [40], we examined if treating ruxolitinib, a JAK1/2-specific inhibitor, could abrogate the protective effect of HHuMin-U on MNV infection. We found that the antiviral activity of HHuMin-U can be largely reversed by ruxolitinib treatment (Fig. 4I). The phosphorylation level of STAT1, which was increased by HHuMin-U treatment in MNV-infected macrophages, decreased when co-treated with ruxolitinib (Fig. 4J). Furthermore, ruxolitinib treatment significantly attenuated the expression levels of HHuMin-U-induced ISGs such as Irf7, Ifit1, and Mda5 (Fig. 4K). These results suggest that the activation of the JAK–STAT signaling pathway and the resulting transcription of ISGs are likely to be the responsible antiviral mechanism of HHuMin-U against MNV.

### Oral administration of HHuMin-U increased IFN-β levels in the small intestine

**Fig. 5.**
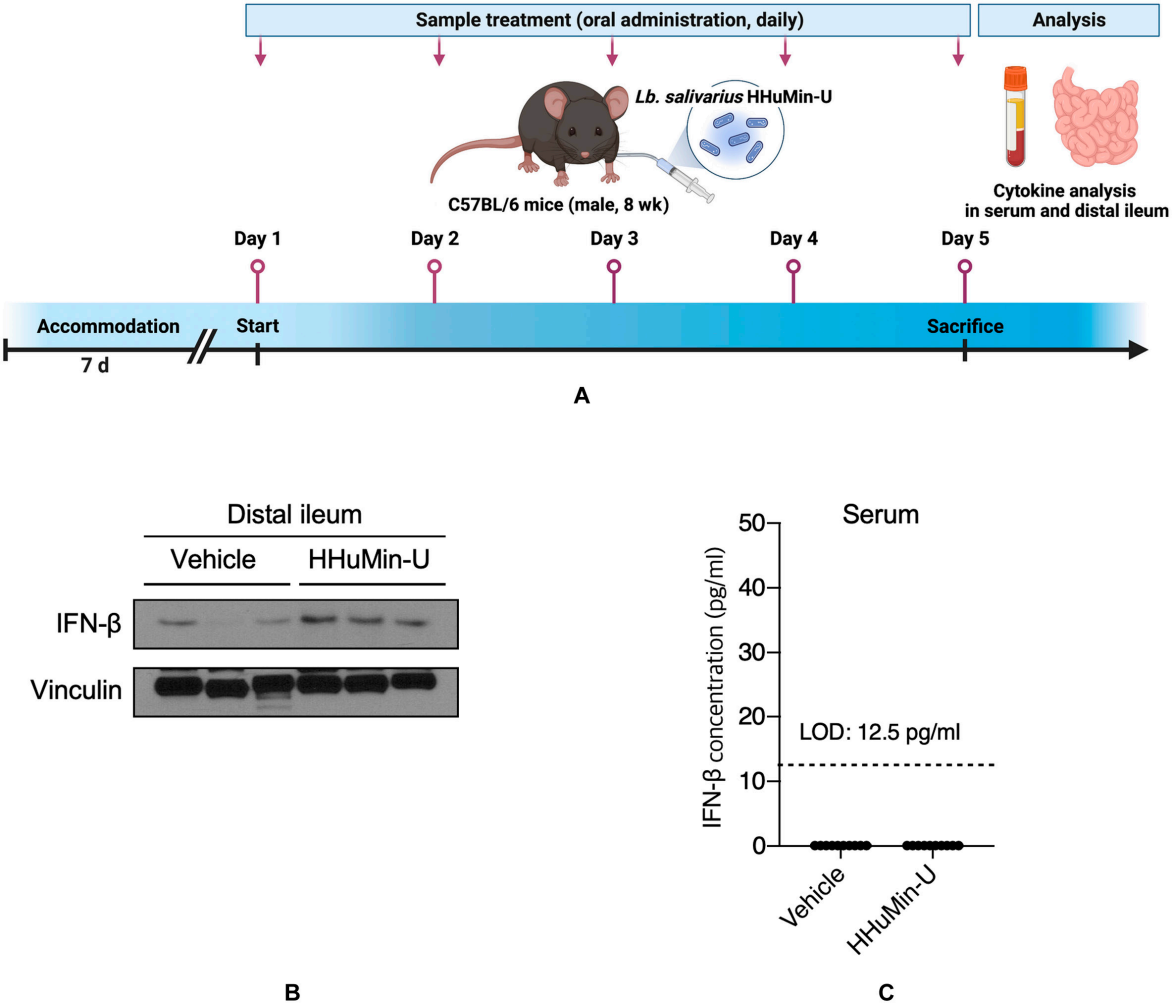
Analysis of IFN-β levels in the ileum tissue and microbiome profiles after the oral administration of HHuMin-U. (A) C57BL/6 mice (8 weeks old, male) were given HHuMin-U (3 × 10^10^ CFU/kg of body weight) once a day for 5 d (created with BioRender.com). The mice were sacrificed, and their ileum and blood samples were harvested on day 5. (B) Immunoblot analysis of the IFN-β expression in ileum tissue extracts. (C) IFN-β expression levels in serum measured by ELISA (*n* = 10 per group).

Noroviruses are reported to target small intestinal tracts, especially the ileum area where they propagate in sentinel cells including macrophages and dendritic cells, and spread to lymph nodes [41,42]. Therefore, we examined whether orally delivered HHuMin-U is capable of inducing IFNs in the ileum part of the small intestine (Fig. 5A). When HHuMin-U (3 × 10^10^ colony-forming units (CFU)/kg of body weight) was administered to mice for 5 d, HHuMin-U-treated mice exhibited increased levels of IFN-β in the ileum tissue compared to vehicle-treated mice (Fig. 5B). However, IFN-β levels in serum were not elevated in the HHuMin-U-treated mice (Fig. 5C), suggesting that HHuMin-U did not trigger systematic inflammatory responses. These results suggest that oral administration of HHuMin-U can display IFN-β-inducing effects in vivo.

### Oral administration of HHuMin-U induces alteration in the gut microbiome

To assess the effect of HHuMin-U supplementation on intestinal bacterial communities, mouse fecal microbiota profiles were analyzed between the vehicle-treated and HHuMin-U-treated mice. The richness and diversity indexes of the microbial community (alpha diversity) in HHuMin-U-treated mice were lower than that in vehicle-treated mice. The significant difference in the Simpson index (control = 0.851 ± 0.077, HHuMin-U = 0.723 ± 0.0.246, *P* = 0.0155; Fig. S2A) was observed. There were no statistically significant differences in the ACE, chao1, and Shannon indexes. The principal coordinates analysis based on Bray–Curtis distance matrix showed a marked separation between 2 groups (Fig. S2B), confirmed by permutational multivariate analysis of variance analysis, indicating a significant difference in beta diversity between the 2 groups (*P* = 0.004, *q* = 0.004)*.*

We totally detected 9 phyla, 13 classes, 41 orders, 67 families, and 152 genera in the gut microbiota community from these 19 feces samples. Relative abundance of the top bacteria at phylum level in vehicle-treated and HHuMin-U-treatment group is the following: *Firmicutes* (80.106% vs. 80.305%), *Bacteroidetes* (9.766% vs. 11.987%), *Actinobacteriota* (5.904% vs. 2.900%), *Verrucomicrobiota* (3.609% vs. 4.651%), and *Cyanobacteria* (0.447% vs. 0%) (Fig. S2C). There was no difference in Firmicutes between the 2 groups. However, *Cyanobacteria* were significantly different (Mann–Whitney U test, *P* = 0.001) (Fig. S2E). Relative abundance of the top bacteria at genus level in vehicle-treated and HHuMin-U-treated group is the following: *Lactobacillus* (49.46% vs. 59.97%), *Bacteroides* (8.15% vs. 11.28%), *Akkermansia* (3.61% vs. 4.65%), *Clostridia_UCG-014+F534* (4.08% vs. 3.08%), *Bifidobacterium* (4.45% vs. 1.69%), *Candidatus_Arthromit+F534* (3.22% vs. 1.94%), *Monoglobus* (2.51% vs. 1.51%), *Lachnospiraceae_NK4A136_group* (1.38% vs. 1.24%), *[Eubacterium]_coprostanoligenes_group* (1.20% vs. 0.73%), *Alistipes* (1.30% vs. 0.42%), *Romboutsia* (0.91% vs. 0.27%), *Enterorhabdus* (0.65% vs. 0.57%), *Turicibacter* (1.08% vs. 0.01%, *P* = 0.012), *Eubacterium]_xylanophilum_group* (0.42% vs. 0.72%) (Fig. S2D)*.* Although the relative abundance of *Lactobacillus* in the HHuMin-U-treated group is higher than the vehicle-treated group, the difference was not statistically significant (*P* = 0.459) (Fig. S2E). However, what is noteworthy is that the relative abundance of *Turicibacter* in the HHuMin-U-treated group was significantly lower than that in the control group (*P* = 0.012) (Fig. S2E). According to a human norovirus challenge study, there was a difference between the microbiome of symptomatic and asymptomatic individuals before the norovirus infection [29]. The microbiomes of symptomatic individuals were enriched with several genera including *Turicibacter* compared to that of asymptomatic individuals, indicating that this genus may increase host susceptibility to symptomatic norovirus infection. This suggests that HHuMin-U treatment might alter the composition of microbiome in a way that enhances host resistance to symptomatic norovirus infection.

The target genes for species specific primer of *Lb. salivarius* between 2 groups were constructed using the QIAcuity digital PCR (dPCR) system for 3.6 × 10^4^ and 9.18 × 10^6^ copies/μl as evaluated by fluorometry, respectively. Fecal microbiota from the HHuMin-U-treated groups had a significantly higher proportion of *Lb. salivarius* based on species-specific dPCR results (*P* = 0.0006; Fig. S2F).

## Discussion

In the current study, we have found that HHuMin-U can exert potent antiviral effects against norovirus infection through upregulating the transcription of diverse antiviral ISGs. HHuMin-U treatment inhibited the replication of MNV and stimulated the production of type I IFNs, IFN-α and IFN-β, and TNF-α. More importantly, oral administration of HHuMin-U locally enhanced IFN-β levels in the intestine, suggesting that the immune-enhancing activity of HHuMin-U can be recapitulated in vivo.

*Lb. salivarius* is a species of lactic acid bacteria, frequently isolated from human digestive tracts or oral cavity [43]. This species has been considered a promising candidate for probiotics, being approved as a safe biological agent for human consumption [44]. Previous works have demonstrated the immunomodulating activity of *Lb. salivarius* spp*.* A study by Ren et al. [45] demonstrated that the *Lb. salivarius* strain promoted immune response by inducing naïve T cell polarization to Th1. Another study reported that oral administration of *Lb. salivarius* B1 enhanced the development of the intestinal mucosal immune system by upregulating the TLR2 expression in the intestinal tract and increasing the number of intestinal immunocompetent cells [46]. In addition, the *Lb. salivarius* UCC118 strain has been shown to upregulate the expression of the pattern recognition receptors such as TLR1 and TLR2 and increase cytokine responses in macrophages [47]. While some of these immune-stimulating efficacies of *Lb. salivarius* strains have been reported, their role in inhibiting virus infections, especially enteric viruses such as norovirus, has not been investigated. Elucidating the antiviral effect of *Lb. salivarius* can be beneficial, considering that noroviruses target enteroendocrine epithelial cells and sentinel cells in gastrointestinal tracts, the sites where these probiotic strains colonize [41]. Especially, a previous work suggested that the relative abundance of the *Lactobacillus* genus in the gut microbiome was significantly decreased after MNV inoculation, suggesting that recovering this gut microbiome abundance might be beneficial [48]. Here, we have demonstrated that *Lb. salivarius* HHuMin-U treatment significantly reduced the viral titer after MNV infection and recovered MNV-induced apoptosis, revealing the antiviral activity of *Lb. salivarius* sp. strain for the first time.

*Lb. salivarius* HHuMin-U was selected as the probiotic strain with the highest antiviral potency through screening a collection of lactic acid bacteria including *Lactobacillus* and *Bifidobacterium* strains. Interestingly, we discovered that the antiviral efficacy varied depending on strains, as *Lb. salivarius* KCTC 43313 showed less IFN-inducible capacity compared to *Lb. salivarius* HHuMin-U (Fig. 1A). Similarly, a previous report has shown that *Lb. plantarum* strains showed different DPP-IV inhibitory activities, resulting in different type 2 diabetes attenuation effects [49]. In addition, specific *Lb. rhamnosus* strains differently modulated cytokine production in human macrophages [50]. These strain-specific discrepancies may be attributed to the differences in molecules present within or at the surface of the probiotic bacterial cells including lipoteichoic acid, CpG-rich DNA motifs, and exopolysaccharides [51–53]. These molecules are reported to be the effector molecules driving diverse probiotic activities of specific strains [54]. Lipoteichoic acid, one of the effector molecules from bacteria is reported to show structural differences depending on the strains [55], which might lead to different efficacies. These results highlight the importance of identifying probiotic bacteria with certain bioactivities at the strain level.

Our data also suggest that HHuMin-U induces the antiviral status of host cells via a type I IFN-dependent mechanism. Type I IFNs play a major role in modulating multiple immune processes to prepare cells into an “antiviral state” [56]. Upon viral infections, IκB kinase-related kinases TBK1 and IKK𝜀 activate the transcription factors such as IRF3 and IRF7, resulting in IFN-β production [39]. The released IFN-β binds to the IFNAR on the cell surface and activates JAK–STAT signaling pathway [57]. Activated tyrosine kinase 2 (TYK2) and JAK1 phosphorylate STAT1 and STAT2. STAT1 and STAT2 interact with IRF9 to form the ISG factor 3 complex, which binds the IFN-stimulated response element promoter and induces the transcription of hundreds of ISGs [58]. These ISGs are ultimate antiviral effectors blocking viral replication. HHuMin-U treatment enhanced the level of type I IFNs (IFN-α and IFN-β) and increased the transcription of various ISGs in macrophages, triggering immune/defense-associated pathways. Suppression of the JAK–STAT pathway using a JAK1/2-specific inhibitor significantly blocked the upregulation of ISGs and attenuated HHuMin-U-mediated inhibition of MNV replication in macrophages. However, other noncanonical pathways might have been involved in the regulation of ISGs by HHuMin-U [59,60].

Type I IFNs and ISGs are reported to establish an antiviral state against numerous viruses including severe acute respiratory syndrome coronavirus 2, influenza, hepatitis C, and hepatitis B infections [61–64], and are considered a therapeutic option to treat chronic virus infection. Indeed, in recent years, type I IFNs, in combination with other drugs, are utilized as a standard treatment for treating hepatitis C and hepatitis B [61]. In addition, ISGs have been shown to participate in inhibiting the replication of important human and animal viruses, including the West Nile virus, HIV-1, chikungunya virus, and vesicular stomatitis virus [35]. These results suggest the potential use of HHuMin-U as a broad-spectrum antiviral therapeutic measure.

**Fig. 6.**
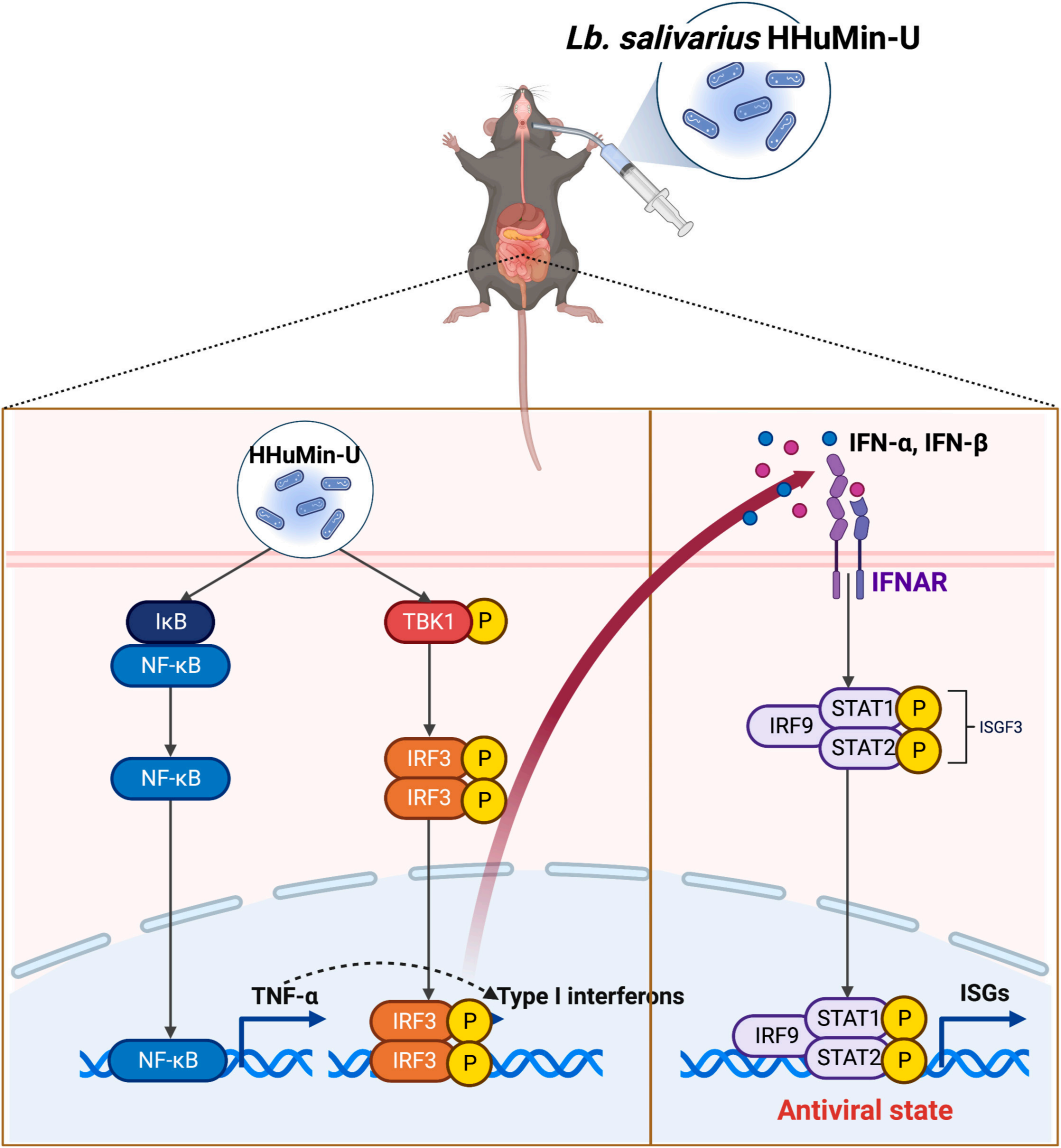
Schematic diagram summarizing the molecular mechanism of HHuMin-U. HHuMin-U activates TBK1–IRF3 and NF-κB signaling, leading to an upregulation of IFN-α, IFN-β, and TNF-α expression. IFN-α and IFN-β induce ISGs via STAT1/2 to enhance antiviral immune response (created with BioRender.com).

In summary, we show that HHuMin-U inhibits the replication of MNV in macrophages; increases type I IFNs, IFN-α and IFN-β, and TNF-α; and upregulates the transcription of diverse ISGs. In addition, we have identified that HHuMin-U activates NF-κB and TBK1–IRF3 signaling pathways, which, in turn, phosphorylates STAT1 and STAT2, the key transcription factors for antiviral ISGs (Fig. 6). Discovering the antiviral efficacy of HHuMin-U will provide a basis for developing HHuMin-U as a probiotic agent for the treatment of norovirus infection.

## Materials and Methods

### Reagents and antibodies

Antibodies to detect IκBα (#4812), p-IκBα (#2859), p-NF-κB (#3033), NF-κB (#8242), p-TBK1 (#5483), TBK1 (#3013), p-IRF3 (#4947), p-STAT1 (#8826), STAT1 (#14995), and STAT2 (#72604) were obtained from Cell Signaling Technology (Danvers, MA, USA). Antibodies to detect IRF-3 (#SC-33641) and β-actin (#SC-47778) were purchased from Santa Cruz Biotechnology Inc. (Dallas, TX, USA). Antibodies to detect p-STAT2 (#07-224) were purchased from Sigma-Aldrich (St. Louis, MO, USA). Ruxolitinib was obtained from SignalChem Biotech Inc. (British Columbia, Canada).

### Preparation of *Lb. salivarius* HHuMin-U

*Lb. salivarius* HHuMin-U was isolated from healthy Korean adults and infants by BIFIDO Co. Ltd. (Hongcheon, Korea). It was identified as *Lb. salivarius* through 16S ribosomal RNA (rRNA) sequencing (Macrogen, Seoul, Korea). HHuMin-U was inoculated at 10% (v/v) in de Man, Rogosa, and Sharpe (BD, USA) broth and grown under anaerobic condition (85% N_2_, 10% H_2_, and 5% CO_2_). The culture was incubated at pH 5.5 and 37 °C with 150 revolutions/min of agitation for 15 h. HHuMin-U was collected by centrifugation (4 °C, 15 min, 10,000 g), and washed with PBS and distilled water. To prepare heat-inactivated HHuMin-U, live HHuMin-U were lyophilized using a Speed-Vac (Ilshin Bio Base, Seoul, Korea), and the freeze-dried cell was resuspended in distilled water and kept at 80 °C for 30 min. HHuMin-U (20 μg/ml) corresponds to 4.12 × 10^5^ CFU/ml.

### Cell culture

RAW264.7 was obtained from the American Type Culture Collection (Manassas, VA, USA). THP-1 was purchased from the Korean Cell Line Bank (Seoul, Korea). RAW264.7 cells were maintained in Dulbecco’s modified Eagle’s media (Welgene, Seoul, Korea) with 10% fetal bovine serum and 1% penicillin and streptomycin. THP-1 cells were cultured in RPMI 1640 (Welgene, Seoul, Korea) supplemented with 10% fetal bovine serum, 0.05 mM 2-mercaptoethanol, and 1% penicillin and streptomycin. THP-1 cells were differentiated with 50 nM phorbol 12-myristate 13-acetate for 3 d.

### Cell viability assay

Sulforhodamine B (SRB)-based (TOX6, Sigma-Aldrich) staining was used to measure the cell viability. The SRB assay is based on the property of SRB dye binding to cellular proteins. Cells were treated with the indicated concentration of HHuMin-U for 48 h. Cells were fixed with cold 50% (w/v) trichloroacetic acid at 4 °C for 1 h and stained with SRB solution for 30 min. The excess dye was rinsed with 1% (v/v) acetic acid, and 10 mM tris base solution was added to dissolve the dye. The absorbance of the dye was measured at an optical density of 554 nm on the Varioskan multimode microplate reader (Thermo Fisher Scientific, MA, USA).

### MNV infection and plaque assay

Infections were carried at a MOI of 0.05. The MNV inoculum was removed, and the fresh complete media with or without HHuMin-U was added back to cells for 24 h. MNV titer was quantified by plaque assay as described previously [65]. RAW264.7 cells were infected with harvested media for 1 h. SeaPlaque Agarose (Lonza, Rockland, ME, USA) in complete Dulbecco’s modified Eagle’s media was overlaid on the cells for 48 h after removing the inoculum. Plaques were visualized with crystal violet staining.

### Enzyme-linked immunosorbent assay (ELISA)

Harvested media were centrifuged to collect supernatants and stored at −80 °C. The concentration of IFN-β and TNF-α were quantified using mouse IFN-β and TNF-α DuoSet enzyme-linked immunosorbent assay (ELISA) kits (R&D Systems Inc., Minneapolis, MN, USA), and the concentration of IFN-α was measured using a mouse IFN-α ELISA kit (PBL Assay Science, NJ, USA) and according to the manufacturer’s protocol. The absorbance was measured with the Varioskan multimode microplate reader (Thermo Fisher Scientific).

### Real-time quantitative polymerase chain reaction (RT-qPCR)

After isolation of RNA, complementary DNA was synthesized using a ReverTra Ace qPCR RT Master Mix with gDNA Remover (TOYOBO, Osaka, Japan). Real-time PCR was performed with CFX Connect Real-Time System (Bio-Rad, Hercules, CA, USA) and SYBR qPCR Mix (TOYOBO, Osaka, Japan). Glyceraldehyde-3-phosphate dehydrogenase (GAPDH) was used as a housekeeping gene to normalize gene expression. The RNA copy number of MNV was quantified as described previously [66]. The specific primer sequences are in Table.

**Table.** Primer sequence for RT-qPCR.

Gene	Primer set	Sequence (5′–3′)
MNV-1	Forward	CACGCCACCGATCTGTTCTG
	Reverse	GCGCTGCGCCATCACTC
mISG15	Forward	TGACGCAGACTGTAGACACG
	Reverse	TGGGGCTTTAGGCCATACTC
mMX1	Forward	GGGGAGGAAATAGAGAAAATGAT
	Reverse	GTTTACAAAGGGCTTGCTTGCT
mIRF7	Forward	CCTCTGCTTTCTAGTGATGCCG
	Reverse	CGTAAACACGGTCTTGCTCCTG
mIFIT1	Forward	CCATAGCGGAGGTGAATATC
	Reverse	GGCAGGACAATGTGCAAGAA
mMDA5	Forward	GTGATGACGAGGCCAGCAGTTG
	Reverse	ATTCATCCGTTTCGTCCAGTTTCA
mCCL2	Forward	AGGTCCCTGTCATGCTTCTG
	Reverse	TCTGGACCCATTCCTTCTTG
mIFN-α	Forward	CCTGTGTGATGCAACAGGTC
	Reverse	TCACTCCTCCTTGCTCAATC
mIFN-β	Forward	AACAGGTGGATCCTCCACGCTGCG
	Reverse	GTGGAGAGCAGTTGAGGACATCTCC
mTNF-α	Forward	TCTTCTCATTCCTGCTTGTGG
	Reverse	GGTCTGGGCCATAGAACTGA
hIFN-α	Forward	AGCCATCTCTGTCCTCCATGA
	Reverse	CATGATTTCTGCTCTGACAACC
hIFN-β	Forward	GAACTTTGACATCCCTGAGGAGATTAAGCAGC
	Reverse	GTTCCTTAGGATTTCCACTCTGACTATGGTCC
hTNF-α	Forward	TCTTCTCGAACCCCGAGTGA
	Reverse	CCTCTGATGGCACCACCAG
mGAPDH	Forward	ACCCAGCCCAGCAAGGATAC
	Reverse	ATTGAGAGGGAGATGCTCAGTGT
hGAPDH	Forward	CAAATTCCATGGCACCGTC
	Reverse	TCTCGCTCCTGGAAGATGGT

### Immunoblotting

Proteins from harvested cells were analyzed by immunoblotting as described previously [67]. Briefly, after protein quantification, equal amounts of cell lysates were separated by SDS-polyacrylamide gel electrophoresis and transferred to a nitrocellulose membrane. The membrane was blocked and incubated with a particular primary antibody. After incubating with a secondary antibody, Western Lightning Plus-ECL (PerkinElmer, Waltham, MA, USA) was used to visualize the protein bands by an automatic x-ray film processor.

### Immunofluorescence staining

For immunostaining, cells were fixed with 4% paraformaldehyde after washing with phosphate-buffered saline (PBS) and then permeabilized with 0.1% Triton X-100. Subsequently, cells were washed twice and blocked in 2% bovine serum albumin in PBS. Cells were incubated with the primary antibody overnight. The next day, coverslips were washed and incubated with an Alexa Fluor 568 antibody (Thermo Fisher Scientific) and then stained with Hoechst 33342. The images were visualized using LSM 900 (Carl Zeiss, Oberkochen, Germany) after mounting the coverslip with mounting media.

### Animal experiments

Animal experiments were approved by the Institutional Animal Care and Use Committee of the Yonsei University (IACUC-202109-1339-01). Eight-week-old male C57BL/6 mice were obtained from Orient Bio Inc. (Sungnam, Korea). Mice were accommodated for 1 week before the experiment under a 12-h light/12-h dark cycle in an air-conditioned room (23 ± 2 °C). Mice were divided into 2 groups: (i) vehicle (PBS)-treated group and (ii) HHuMin-U (3 × 10^10^ CFU/kg of body weight)-treated group. Vehicle (PBS) or HHuMin-U was orally administered to mice once a day for 5 d through a metal gastric zonde. On day 5, mice were anesthetized with CO_2_ and sacrificed for analysis of serum, ileum, and gut microbiome.

### Transcriptome sequencing analysis

RAW264.7 cells were treated with HHuMin-U for 12 h, and total RNA was isolated from cells by RNeasy Mini kit (QIAGEN, Hilden, Germany). 2100 Bioanalyzer (Agilent Technologies, Palo Alto, CA, USA) was used to determine the integrity of the RNA before proceeding with the downstream analysis. Complementary DNA libraries were constructed using the TruSeq Stranded mRNA LT Sample Prep Kit (Illumina, San Diego, CA, USA) according to the manufacturer’s protocol. Libraries were validated running on a 2100 Bioanalyzer before sequencing 100-base pair paired-end reads on the Illumina sequencing platforms. GO enrichment was performed using gProfileR, and all statistical analyses and data visualization were done in R software using R basic functions and the following packages: ggplot2, heatmap.2, and stats.

### Genomic DNA extraction and 16S rRNA gene sequencing

Total bacterial genomic DNA was isolated using the MagMAX Microbiome Ultra Nucleic Acid Isolation Kit on KingFisher Flex automated DNA/RNA isolation system (Thermo Fisher Scientific), according to the manufacturer’s instructions. 16S rRNA gene sequencing was performed following the Illumina 16S Metagenomic Sequencing Library preparation guide protocol (Illumina). The variable regions 3 and 4 of the 16S rRNA gene were amplified from the genomic DNA of feces samples. The 16S rRNA sequence was amplified as described previously [68]. Each PCR product was purified using the Agencourt AMPure XP purification system (Beckman Coulter, Brea, CA, USA). The library was quantified and estimated in size using the QIAxcel Advanced with QIAxcel DNA High-Resolution Kit (QIAGEN, Hilden, Germany). Amplicons were pooled using the Illumina MiSeq System (2× 300-base pair paired-end reads, Illumina, USA).

### Bioinformatic analysis of sequencing data

The sequence assembly and quality filtering on the raw tags were processed via QIIME2 software (version 2020.8; https://docs.qiime2.org/2020.8/). In QIIME2, we followed the “Moving Pictures” tutorial version 2020.08. Raw sequences were demultiplexed using the q2-demux plugin. Then, the DADA2 plugin was used to denoise the sequences with the low-quality score. All amplicon sequence variants were classified against the SILVA 138 99% database. QIIME 2’s diversity analyses were also performed using the q2-diversity plugin (“core-metrics-phylogenetic,” “alpha-group-significance,” and “beta-group-significance”). R software was also applied to display microbial richness (ACE and Chao1), diversity (Shannon and Simpson) indexes and the principal coordinate analysis.

### Bioinformatic analysis of sequencing data

QIAcuity dPCR (QIAGEN) is performed on a microfluidic QIAcuity nanoplate (QIAGEN) according to the manufacturer’s instructions. The partitions for each well are imaged, and data analysis is carried out using the QIAcuity Software Suite (QIAGEN) after thermocycling. The Mann–Whitney U test using GraphPad Prism Software (v 9.0) (GraphPad Software, San Diego, CA, USA) was performed to determine the significant differences in microbial diversity. Permutational multivariate analysis of variance was utilized in the QIIME2 plugin to assess the difference in community structure.

### Statistical analysis

All the experimental data are presented as mean ± SD. For statistical analysis, 2-trailed Student’s *t* test was used, and *P* values of less than 0.05 were considered statistically significant unless otherwise stated. All statistical analyses were performed using GraphPad Prism Software (version 9.0).

## Data Availability

All data used to evaluate the findings in the paper are present in the paper and/or the Supplementary Materials. Raw RNA-seq data are available upon request.

## Supplementary Materials

Supplementary Materials Fig. S1. mRNA expression levels of type I IFNs, IFN-α and IFN-β, and TNF-α in human macrophage-like cells. Fig. S2. Microbiome profiles after the oral administration of HHuMin-U.

## References

[1] Bartsch SM, Lopman BA, Ozawa S, Hall AJ, Lee BY. Global economic burden of norovirus gastroenteritis. PLOS ONE. 2016;11(4):e0151219. 27115736 10.1371/journal.pone.0151219 PMC4846012

[2] Pires SM, Fischer-Walker CL, Lanata CF, Devleesschauwer B, Hall AJ, Kirk MD, Duarte AS, Black RE, Angulo FJ. Aetiology-specific estimates of the global and regional incidence and mortality of diarrhoeal diseases commonly transmitted through food. PLOS ONE. 2015;10(12):e0142927. 26632843 10.1371/journal.pone.0142927 PMC4668836

[3] Cho HG, Lee SG, Lee MY, Hur ES, Lee JS, Park PH, Park YB, Yoon MH, Paik SY. An outbreak of norovirus infection associated with fermented oyster consumption in South Korea, 2013. Epidemiol Infect. 2016;144(13):2759–2764. 26830365 10.1017/S0950268816000170 PMC9150470

[4] Guo Z, Huang J, Shi G, Su CH, Niu JJ. A food-borne outbreak of gastroenteritis caused by norovirus GII in a university located in Xiamen City, China. Int J Infect Dis. 2014;28:101–106. 25263502 10.1016/j.ijid.2014.06.022

[5] Glass RI, Parashar UD, Estes MK. Norovirus gastroenteritis. N Engl J Med. 2009;361(18):1776–1785. 19864676 10.1056/NEJMra0804575 PMC3880795

[6] Grima A, Gatt A, Zahra G, Gambin A. Outbreak of norovirus infection in a nursing home for the elderly in Malta, November-December 2008. Euro Surveill. 2009;14(4):19103. 19215711

[7] Loury P, Le Guyader FS, Le Saux JC, Ambert-Balay K, Parrot P, Hubert B. A norovirus oyster-related outbreak in a nursing home in France, January 2012. Epidemiol Infect. 2015;143(12):2486–2493. 25567093 10.1017/S0950268814003628 PMC9151012

[8] Khanna N, Goldenberger D, Graber P, Battegay M, Widmer AF. Gastroenteritis outbreak with norovirus in a Swiss university hospital with a newly identified virus strain. J Hosp Infect. 2003;55(2):131–136. 14529638 10.1016/s0195-6701(03)00257-3

[9] Isakbaeva ET, Widdowson MA, Beard RS, Bulens SN, Mullins J, Monroe SS, Bresee J, Sassano P, Cramer EH, Glass RI. Norovirus transmission on cruise ship. Emerg Infect Dis. 2005;11(1):154–158. 15705344 10.3201/eid1101.040434 PMC3294347

[10] Green KY. Norovirus infection in immunocompromised hosts. Clin Microbiol Infect. 2014;20(8):717–723. 25040790 10.1111/1469-0691.12761 PMC11036326

[11] Munir N, Liu P, Gastañaduy P, Montes J, Shane A, Moe C. Norovirus infection in immunocompromised children and children with hospital-acquired acute gastroenteritis. J Med Virol. 2014;86(7):1203–1209. 24115094 10.1002/jmv.23774

[12] Murira A, Lamarre A. Type-I interferon responses: From friend to foe in the battle against chronic viral infection. Front Immunol. 2016;7:609. 28066419 10.3389/fimmu.2016.00609 PMC5165262

[13] Karst SM, Wobus CE, Lay M, Davidson J, Virgin HW IV. STAT1-dependent innate immunity to a Norwalk-like virus. Science. 2003;299(5612):1575–1578. 12624267 10.1126/science.1077905

[14] Thackray LB, Duan E, Lazear HM, Kambal A, Schreiber RD, Diamond MS, Virgin HW. Critical role for interferon regulatory factor 3 (IRF-3) and IRF-7 in type I interferon-mediated control of murine norovirus replication. J Virol. 2012;86(24):13515–13523. 23035219 10.1128/JVI.01824-12 PMC3503103

[15] Nice TJ, Osborne LC, Tomov VT, Artis D, Wherry EJ, Virgin HW. Type I interferon receptor deficiency in dendritic cells facilitates systemic murine norovirus persistence despite enhanced adaptive immunity. PLOS Pathog. 2016;12(6):e1005684. 27327515 10.1371/journal.ppat.1005684 PMC4915689

[16] Davis A, Cortez V, Grodzki M, Dallas R, Ferrolino J, Freiden P, Maron G, Hakim H, Hayden RT, Tang L, et al. Infectious norovirus is chronically shed by immunocompromised pediatric hosts. Viruses. 2020;12(6):619. 32516960 10.3390/v12060619 PMC7354526

[17] Koo HL, DuPont HL. Noroviruses as a potential cause of protracted and lethal disease in immunocompromised patients. Clin Infect Dis. 2009;49(7):1069–1071. 19705972 10.1086/605558

[18] Woodward JM, Gkrania-Klotsas E, Cordero-Ng AY, Aravinthan A, Bandoh BN, Liu H, Davies S, Zhang H, Stevenson P, Curran MD, et al. The role of chronic norovirus infection in the enteropathy associated with common variable immunodeficiency. Am J Gastroenterol. 2015;110(2):320–327. 25623655 10.1038/ajg.2014.432

[19] Mboko WP, Chhabra P, Valcarce MD, Costantini V, Vinjé J. Advances in understanding of the innate immune response to human norovirus infection using organoid models. J Gen Virol. 2022;103(1):001720. 10.1099/jgv.0.001720 PMC8984994 35077345

[20] Lin SC, Qu L, Ettayebi K, Crawford SE, Blutt SE, Robertson MJ, Zeng XL, Tenge VR, Ayyar BV, Karandikar UC, et al. Human norovirus exhibits strain-specific sensitivity to host interferon pathways in human intestinal enteroids. Proc Natl Acad Sci USA. 2020;117(38):23782–23793. 32907944 10.1073/pnas.2010834117 PMC7519316

[21] Hosmillo M, Chaudhry Y, Nayak K, Sorgeloos F, Koo BK, Merenda A, Lillestol R, Drumright L, Zilbauer M, Goodfellow I. Norovirus replication in human intestinal epithelial cells is restricted by the interferon-induced JAK/STAT signaling pathway and RNA polymerase II-mediated transcriptional responses. mBio. 2020;11(2):e00215-20. 32184238 10.1128/mBio.00215-20 PMC7078467

[22] Akira S. Pathogen recognition by innate immunity and its signaling. Proc Jpn Acad Ser B Phys Biol Sci. 2009;85(4):143–156. 10.2183/pjab.85.143 PMC3524297 19367086

[23] Moreau MC, Corthier G. Effect of the gastrointestinal microflora on induction and maintenance of oral tolerance to ovalbumin in C3H/HeJ mice. Infect Immun. 1988;56(10):2766–2768. 3417356 10.1128/iai.56.10.2766-2768.1988 PMC259643

[24] Dessein R, Gironella M, Vignal C, Peyrin-Biroulet L, Sokol H, Secher T, Lacas-Gervais S, Gratadoux JJ, Lafont F, Dagorn JC, et al. Toll-like receptor 2 is critical for induction of Reg3 beta expression and intestinal clearance of Yersinia pseudotuberculosis. Gut. 2009;58(6):771–776. 19174417 10.1136/gut.2008.168443

[25] Asquith MJ, Boulard O, Powrie F, Maloy KJ. Pathogenic and protective roles of MyD88 in leukocytes and epithelial cells in mouse models of inflammatory bowel disease. Gastroenterology. 2010;139(2):519-29, 529 e1-2. 20433840 10.1053/j.gastro.2010.04.045 PMC3739016

[26] Majamaa H, Isolauri E, Saxelin M, Vesikari T. Lactic acid bacteria in the treatment of acute rotavirus gastroenteritis. J Pediatr Gastroenterol Nutr. 1995;20(3):333–338. 7608829 10.1097/00005176-199504000-00012

[27] Maragkoudakis PA, Chingwaru W, Gradisnik L, Tsakalidou E, Cencic A. Lactic acid bacteria efficiently protect human and animal intestinal epithelial and immune cells from enteric virus infection. Int J Food Microbiol. 2010;141(Suppl 1):S91–S97. 20106541 10.1016/j.ijfoodmicro.2009.12.024 PMC7114074

[28] Xiong L, Li Y, Li J, Yang J, Shang L, He X, Liu L, Luo Y, Xie X. Intestinal microbiota profiles in infants with acute gastroenteritis caused by rotavirus and norovirus infection: A prospective cohort study. Int J Infect Dis. 2021;111:76–84. 34411719 10.1016/j.ijid.2021.08.024

[29] Patin NV, Peña-Gonzalez A, Hatt JK, Moe C, Kirby A, Konstantinidis KT. The role of the gut microbiome in resisting norovirus infection as revealed by a human challenge study. mBio. 2020;11(6):e02634-20. 33203758 10.1128/mBio.02634-20 PMC7683401

[30] Ibrahim SA, Ayivi RD, Zimmerman T, Siddiqui SA, Altemimi AB, Fidan H, Esatbeyoglu T, Bakhshayesh RV. Lactic acid bacteria as antimicrobial agents: Food safety and microbial food spoilage prevention. Foods. 2021;10(12):3131. 34945682 10.3390/foods10123131 PMC8701396

[31] Grandy G, Medina M, Soria R, Terán CG, Araya M. Probiotics in the treatment of acute rotavirus diarrhoea. A randomized, double-blind, controlled trial using two different probiotic preparations in Bolivian children. BMC Infect Dis. 2010;10:253. 20735858 10.1186/1471-2334-10-253 PMC2940902

[32] Lv XC, Chen M, Huang ZR, Guo WL, Ai LZ, Bai WD, Yu XD, Liu YL, Rao PF, Ni L. Potential mechanisms underlying the ameliorative effect of Lactobacillus paracasei FZU103 on the lipid metabolism in hyperlipidemic mice fed a high-fat diet. Food Res Int. 2021;139:109956. 33509508 10.1016/j.foodres.2020.109956

[33] Li L, Fang Z, Liu X, Hu W, Lu W, Lee YK, Zhao J, Zhang H, Chen W. Lactobacillus reuteri attenuated allergic inflammation induced by HDM in the mouse and modulated gut microbes. PLOS ONE. 2020;15(4):e0231865. 32315360 10.1371/journal.pone.0231865 PMC7173794

[34] Bok K, Prikhodko VG, Green KY, Sosnovtsev SV. Apoptosis in murine norovirus-infected RAW264.7 cells is associated with downregulation of survivin. J Virol. 2009;83(8):3647–3656. 19211757 10.1128/JVI.02028-08 PMC2663291

[35] Schoggins JW, Wilson SJ, Panis M, Murphy MY, Jones CT, Bieniasz P, Rice CM. A diverse range of gene products are effectors of the type I interferon antiviral response. Nature. 2011;472(7344):481–485. 21478870 10.1038/nature09907 PMC3409588

[36] Wu R, Chen F, Wang N, Tang D, Kang R. ACOD1 in immunometabolism and disease. Cell Mol Immunol, 2020. 17(8):822–833. 32601305 10.1038/s41423-020-0489-5 PMC7395145

[37] Metz P, Dazert E, Ruggieri A, Mazur J, Kaderali L, Kaul A, Zeuge U, Windisch MP, Trippler M, Lohmann V, et al. Identification of type I and type II interferon-induced effectors controlling hepatitis C virus replication. Hepatology. 2012;56(6):2082–2093. 22711689 10.1002/hep.25908

[38] Yarilina A, Park-Min KH, Antoniv T, Hu X, Ivashkiv LB. TNF activates an IRF1-dependent autocrine loop leading to sustained expression of chemokines and STAT1-dependent type I interferon-response genes. Nat Immunol. 2008;9(4):378–387. 18345002 10.1038/ni1576

[39] Honda K, Takaoka A, Taniguchi T. Type I inteferon gene induction by the interferon regulatory factor family of transcription factors. Immunity. 2006;25(3):349-360. 16979567 10.1016/j.immuni.2006.08.009

[40] McNab F, Mayer-Barber K, Sher A, Wack A, O'Garra A. Type I interferons in infectious disease. Nat Rev Immunol. 2015;15(2):87–103. 25614319 10.1038/nri3787 PMC7162685

[41] Green KY, Kaufman SS, Nagata BM, Chaimongkol N, Kim DY, Levenson EA, Tin CM, Yardley AB, Johnson JA, Barletta ABF, et al. Human norovirus targets enteroendocrine epithelial cells in the small intestine. Nat Commun. 2020;11(1):2759. 32488028 10.1038/s41467-020-16491-3 PMC7265440

[42] Karst SM, Wobus CE. A working model of how noroviruses infect the intestine. PLOS Pathogens. 2015;11(2):e1004626. 25723501 10.1371/journal.ppat.1004626 PMC4344369

[43] Messaoudi S, Manai M, Kergourlay G, Prévost H, Connil N, Chobert JM, Dousset X. Lactobacillus salivarius: Bacteriocin and probiotic activity. Food Microbiol. 2013;36(2):296–304. 24010610 10.1016/j.fm.2013.05.010

[44] Chaves BD, Brashears MM, Nightingale KK. Applications and safety considerations of Lactobacillus salivarius as a probiotic in animal and human health. J Appl Microbiol. 2017;123(1):18–28. 28256040 10.1111/jam.13438

[45] Ren DY, Wang D, Liu H, Shen M, Yu H. Two strains of probiotic *Lactobacillus* enhance immune response and promote naive T cell polarization to Th1. Food Agric Immunol. 2019;30(1):281–295.

[46] Zhang J, Deng J, Wang Z, Che C, Li YF, Yang Q. Modulatory effects of *Lactobacillus salivarius* on intestinal mucosal immunity of piglets. Curr Microbiol. 2011;62(5):1623–1631. 21365445 10.1007/s00284-011-9906-4

[47] Udayan S, Buttó LF, Rossini V, Velmurugan J, Martinez-Lopez M, Sancho D, Melgar S, O'Toole PW, Nally K. Macrophage cytokine responses to commensal Gram-positive Lactobacillus salivarius strains are TLR2-independent and Myd88-dependent. Sci Rep. 2021;11(1):5896. 33723368 10.1038/s41598-021-85347-7 PMC7961041

[48] Lee H, Ko G. Antiviral effect of vitamin A on norovirus infection via modulation of the gut microbiome. Sci Rep. 2016;6:25835. 27180604 10.1038/srep25835 PMC4867650

[49] Yan F, Li N, Yue Y, Wang C, Zhao L, Evivie SE, Li B, Huo G. Screening for potential novel probiotics with dipeptidyl peptidase IV-inhibiting activity for Type 2 diabetes attenuation *in vitro* and *in vivo*. Front Microbiol. 2019;10:2855. 31998245 10.3389/fmicb.2019.02855 PMC6965065

[50] Rocha-Ramirez LM, Pérez-Solano RA, Castañón-Alonso SL, Moreno Guerrero SS, Ramírez Pacheco A, García Garibay M, Eslava C. Probiotic *Lactobacillus* strains stimulate the inflammatory response and activate human macrophages. J Immunol Res. 2017;2017:4607491. 28758133 10.1155/2017/4607491 PMC5516745

[51] Duboux S, Van Wijchen M, Kleerebezem M. The possible link between manufacturing and probiotic efficacy; a molecular point of view on *Bifidobacterium*. Front Microbiol. 2021;12:812536. 35003044 10.3389/fmicb.2021.812536 PMC8741271

[52] Lebeer S, Verhoeven TL, Francius G, Schoofs G, Lambrichts I, Dufrêne Y, Vanderleyden J, De Keersmaecker SC. Identification of a gene cluster for the biosynthesis of a long, galactose-rich Exopolysaccharide in *Lactobacillus rhamnosus* GG and functional analysis of the priming glycosyltransferase. Appl Environ Microbiol. 2009;75(11):3554–3563. 19346339 10.1128/AEM.02919-08 PMC2687306

[53] Lebeer S, Claes IJ, Vanderleyden J. Anti-inflammatory potential of probiotics: Lipoteichoic acid makes a difference. Trends Microbiol. 2012;20(1):5–10. 22030243 10.1016/j.tim.2011.09.004

[54] Lebeer S, Bron PA, Marco ML, Van Pijkeren JP, O'Connell Motherway M, Hill C, Pot B, Roos S, Klaenhammer T. Identification of probiotic effector molecules: Present state and future perspectives. Curr Opin Biotechnol. 2018;49:217–223. 29153882 10.1016/j.copbio.2017.10.007

[55] Reichmann NT, Grundling A. Location, synthesis and function of glycolipids and polyglycerolphosphate lipoteichoic acid in Gram-positive bacteria of the phylum Firmicutes. FEMS Microbiol Lett. 2011;319(2):97–105. 21388439 10.1111/j.1574-6968.2011.02260.x PMC3089915

[56] Pestka S, Krause CD, Walter MR. Interferons, interferon-like cytokines, and their receptors. Immunol Rev. 2004;202:8–32. 15546383 10.1111/j.0105-2896.2004.00204.x

[57] Malmgaard L. Induction and regulation of IFNs during viral infections. J Interferon Cytokine Res. 2004;24(8):439–54. 15320958 10.1089/1079990041689665

[58] Chathuranga K, Weerawardhana A, Dodantenna N, Lee JS. Regulation of antiviral innate immune signaling and viral evasion following viral genome sensing. Exp Mol Med. 2021;53(11):1647–1668. 34782737 10.1038/s12276-021-00691-y PMC8592830

[59] Wang W, Xu L, Su J, Peppelenbosch MP, Pan Q. Transcriptional regulation of antiviral interferon-stimulated genes. Trends Microbiol. 2017;25(7):573–584. 28139375 10.1016/j.tim.2017.01.001 PMC7127685

[60] Xu L, Wang W, Peppelenbosch MP, Pan Q. Noncanonical antiviral mechanisms of ISGs: Dispensability of inducible interferons. Trends Immunol. 2017;38(1):1–2. 27916385 10.1016/j.it.2016.11.002

[61] Lin FC, Young HA. Interferons: Success in anti-viral immunotherapy. Cytokine Growth Factor Rev. 2014;25(4):369–376. 25156421 10.1016/j.cytogfr.2014.07.015 PMC4182113

[62] Kim YM, Shin EC. Type I and III interferon responses in SARS-CoV-2 infection. Exp Mol Med. 2021;53(5):750–760. 33953323 10.1038/s12276-021-00592-0 PMC8099704

[63] Wu W, Metcalf JP. The role of type I IFNs in influenza: Antiviral superheroes or immunopathogenic villains? J Innate Immun. 2020;12(6):437–447. 32564033 10.1159/000508379 PMC7747089

[64] Ye J, Chen J. Interferon and hepatitis B: Current and future perspectives. Front Immunol. 2021;12:733364. 34557195 10.3389/fimmu.2021.733364 PMC8452902

[65] Gonzalez-Hernandez MB, Bragazzi Cunha J, Wobus CE. Plaque assay for murine norovirus. J Vis Exp. 2012;66:e4297. 10.3791/4297 PMC3487293 22951568

[66] Kageyama T, Kojima S, Shinohara M, Uchida K, Fukushi S, Hoshino FB, Takeda N, Katayama K. Broadly reactive and highly sensitive assay for Norwalk-like viruses based on real-time quantitative reverse transcription-PCR. J Clin Microbiol. 2003;41(4):1548–1557. 12682144 10.1128/JCM.41.4.1548-1557.2003 PMC153860

[67] Shin SH, Lee JS, Zhang JM, Choi S, Boskovic ZV, Zhao R, Song M, Wang R, Tian J, Lee MH, et al. Synthetic lethality by targeting the RUVBL1/2-TTT complex in mTORC1-hyperactive cancer cells. Sci Adv. 2020;6(31):eaay9131. 32789167 10.1126/sciadv.aay9131 PMC7399646

[68] Kim H, Kim S, Park S, Park G, Shin H, Park MS, Kim J. Administration of *Bifidobacterium bifidum* BGN4 and *Bifidobacterium longum* BORI improves cognitive and memory function in the mouse model of Alzheimer's Disease. Front Aging Neurosci. 2021;13:709091. 34421576 10.3389/fnagi.2021.709091 PMC8378450

