# Supplementary material for: Lactobacillus salivarius HHuMin-U Activates Innate Immune Defense against Norovirus Infection through TBK1-IRF3 and NF-κB Signaling Pathways
Source: Research (Wash D C). 2022 Dec 19;2022:0007. doi: 10.34133/research.0007 (PMC11407524; doi:10.34133/research.0007)
Supplement: Supplementary Materials — Fig. S1. mRNA expression levels of type I IFNs, IFN-α and IFN-β, and TNF-α in human macrophage-like cells. Fig. S2. Microbiome profiles after the oral administration of HHuMin-U. [file research.0007.f1.docx]

Supplementary Materials

***Lactobacillus salivarius* HHuMin-U activates innate immune defense against norovirus infection through TBK1-IRF3 and NF-κB signaling pathways**

Da Hyun Kim^1^, Minju Jeong^2^, Jae Hwan Kim^1^, Joe Eun Son^3^, John J. Y. Lee^3^, Sang-jun Park^4^, Juyeon Lee^4^, Minwoo Kim^1^, Jong-Won Oh^1^, Myeong Soo Park^4^, and Sanguine Byun^1,*^

^1^Department of Biotechnology, Yonsei University, Seoul 03722, Republic of Korea

^2^Department of Agricultural Biotechnology, Seoul National University, Seoul 08826, Republic of Korea

^3^Program in Developmental & Stem Cell Biology, The Hospital for Sick Children, Toronto, Ontario, Canada

^4^Research Center, BIFIDO Co., Ltd., Hanam 12930, Republic of Korea

*Corresponding author: Sanguine Byun (Email: [sanguine@yonsei.ac.kr](mailto:sanguine@yonsei.ac.kr); Phone: +82-2-2123-5896)

**
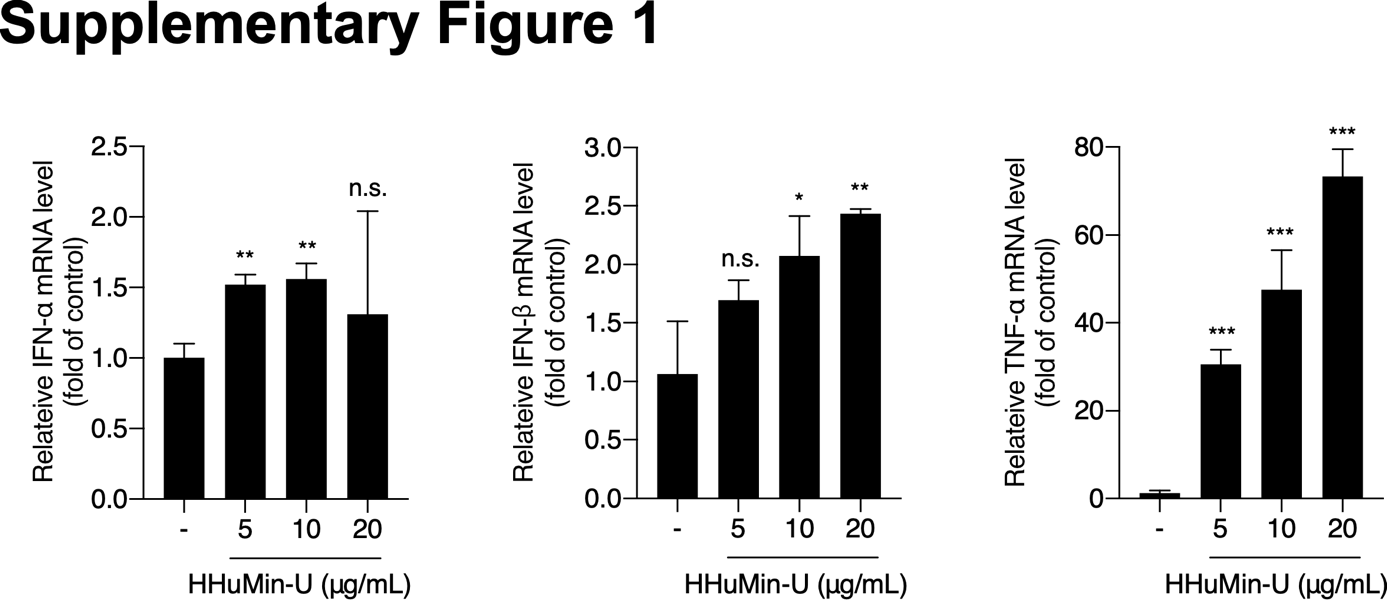
**

**Supplementary Figure 1. mRNA expression levels of IFN-α, IFN-β, and TNF-α in human macrophage-like cells.**

The cells were treated with increasing concentrations of HHuMin-U or LPS (200 ng/mL) for 24 h. The mRNA expression levels of IFN-α, IFN-β, and TNF-α in THP-1 cells were determined using RT-qPCR. Data are shown as mean ± S.D. * *p* < 0.05; ** *p* < 0.01; *** *p* < 0.001; n.s., not significant.


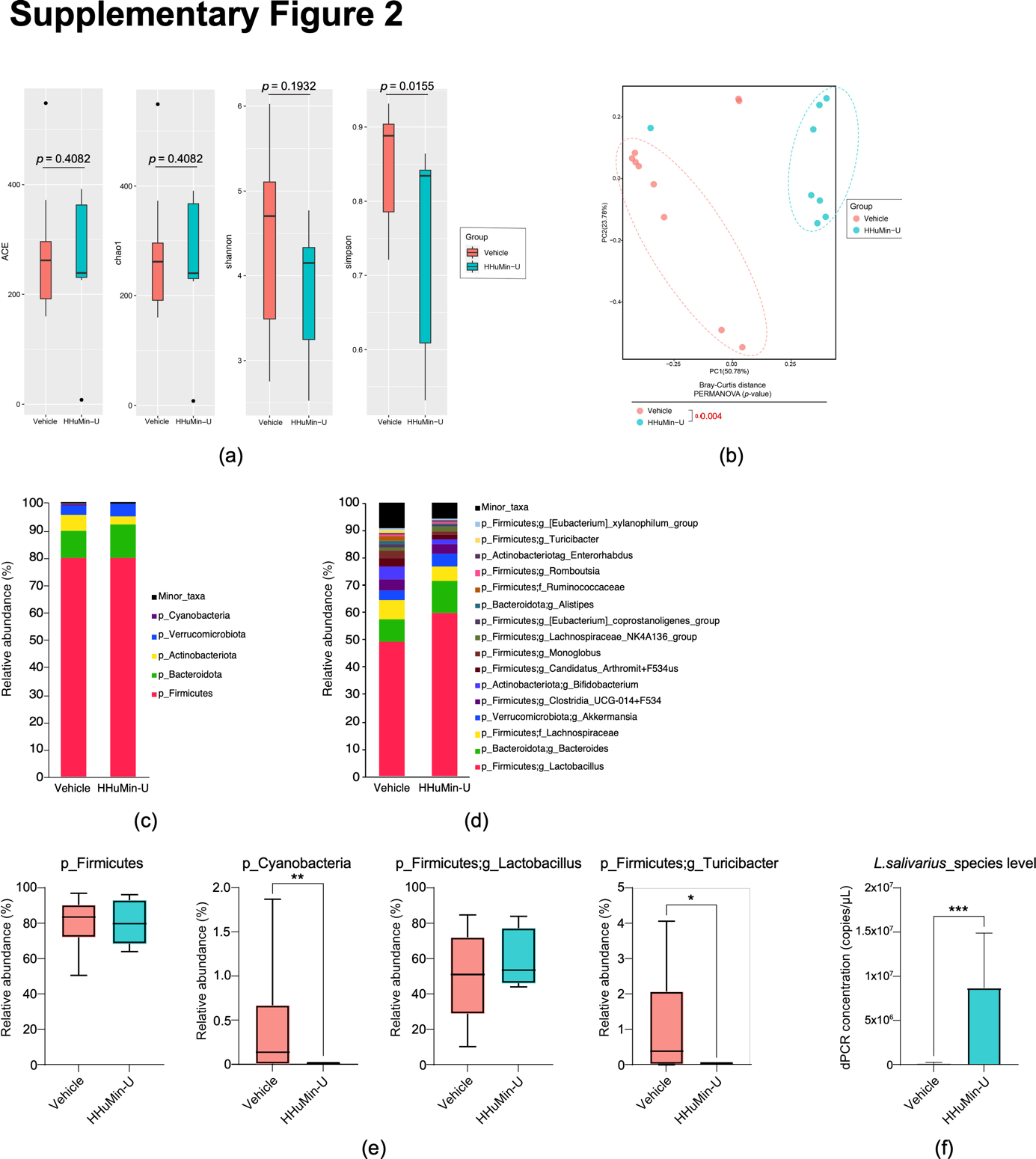


**Supplementary Figure 2. Microbiome profiles after the oral administration of HHuMin-U.**

(a) Microbial alpha-diversity. Microbial richness (ACE, Chao1) and diversity (shannon, simpson) indexes are shown. (b) Microbial beta-diversity. A principal coordinate analysis (PCoA) of the Bray-Curtis dissimilarity index of samples is shown. (c) Changes in microbial composition at Phylum level between groups. Other represents all genera not in the bottom 0.5%. (d) Changes in microbial composition at Genus level between groups. Other represents all genera not in the bottom 0.5%. (e) Different patterns are exhibited in bacterial phylum and genes between groups. (f) The quantification of target DNA was calculated using QIAcuity dPCR System and presented as copy numbers per 12 μL of the reaction mixture. Data are shown as mean ± S.D. **p* < 0.05, ** *p* < 0.01, *** *p* < 0.001 (Mann–Whitney U test).
